# The testicular microvasculature in Klinefelter syndrome is immature with compromised integrity and characterized by excessive inflammatory cross-talk

**DOI:** 10.1093/humrep/dead224

**Published:** 2023-10-31

**Authors:** Emma B Johannsen, Anne Skakkebæk, Joanna M Kalucka, Jens Fedder, Claus H Gravholt, Jesper Just

**Affiliations:** Department of Molecular Medicine, Aarhus University Hospital, Aarhus N, Denmark; Department of Clinical Medicine, Aarhus University, Aarhus N, Denmark; Department of Molecular Medicine, Aarhus University Hospital, Aarhus N, Denmark; Department of Clinical Medicine, Aarhus University, Aarhus N, Denmark; Department of Clinical Genetics, Aarhus University Hospital, Aarhus N, Denmark; Department of Biomedicine, Aarhus University, Aarhus C, Denmark; Steno Diabetes Center Aarhus, Aarhus University Hospital, Aarhus N, Denmark; Centre of Andrology and Fertility Clinic, Odense University Hospital, Odense C, Denmark; Research Unit of Gynaecology and Obstetrics, University of Southern Denmark, Odense C, Denmark; Department of Molecular Medicine, Aarhus University Hospital, Aarhus N, Denmark; Department of Clinical Medicine, Aarhus University, Aarhus N, Denmark; Department of Endocrinology, Aarhus University Hospital, Aarhus N, Denmark; Department of Molecular Medicine, Aarhus University Hospital, Aarhus N, Denmark; Department of Clinical Medicine, Aarhus University, Aarhus N, Denmark

**Keywords:** Klinefelter syndrome, testes, microvasculature, hypogonadism, azoospermia, single-cell RNA sequencing

## Abstract

**STUDY QUESTION:**

Does Klinefelter syndrome (KS) lead to a distinct gene expression pattern at single-cell level in the testes that could provide insight into the reported microvascular dysfunction in the testes?

**SUMMARY ANSWER:**

A distinct gene expression pattern within microvascular-associated cells of males with KS suggests excessive endothelial cell (EC) activation, disorganized vessel formation, and the presence of immature vessels with compromised integrity.

**WHAT IS KNOWN ALREADY:**

Recent studies show that males with KS exhibit microvascular dysfunction in their testes, which affects blood flow and is associated with lower circulating levels of testosterone.

**STUDY DESIGN, SIZE, DURATION:**

A comparative cross-sectional study of males with KS (n = 6), non-obstructive azoospermia (NOA) (n = 5), cryptozoospermia (n = 3), and controls (n = 15) was carried out.

**PARTICIPANTS/MATERIALS, SETTING, METHODS:**

We analyzed publicly available single-cell RNA sequencing data of testicular cells from males with KS, males with NOA, males with cryptozoospermia, and controls. The integration of these datasets allowed us to analyze gene expression profiles and communication patterns among the cell types within the testis and to identify capillary ECs to investigate changes at the microvascular level.

**MAIN RESULTS AND THE ROLE OF CHANCE:**

Rooted in changes at the single-cell level, our study demonstrates a shift in gene expression forming the foundation for altered cellular communication, microvascular remodeling, and pro-inflammatory responses within the testes of males with KS. We identified genes that were dysregulated in capillary ECs from males with KS (*P*_adj_ < 0.05). Specifically, the unique microvascular gene expression in males with KS indicated enhanced capillary EC activation and increased inflammatory cross-talk, leading to impaired vessel maturation and increased EC barrier permeability.

**LIMITATIONS, REASONS FOR CAUTION:**

Our study is constrained by an unbalanced design, with varying sample sizes and number of cells within each group. We acknowledge the restricted access to clinical information. In addition, our findings were deduced from changes in gene expression, which limits us to infer potential biological consequences arising from these alterations. Furthermore, the absence of a pre-pubertal age group limits the generalizability of our findings and warrants further investigation.

**WIDER IMPLICATIONS OF THE FINDINGS:**

This study offers novel insights into the testicular pathophysiology in KS and underscores the potential contribution of microvascular dysfunction to the hypogonadism and infertility observed in males with KS. While this study aims to better understand the microvascular dysfunction in KS, the precise connections to testosterone deficiency and testicular atrophy remain to be fully elucidated.

**STUDY FUNDING/COMPETING INTEREST(S):**

A.S. was supported by the Independent Research Fund Denmark (0134-00130B). C.H.G. was supported by Novo Nordisk Foundation (NNF15OC0016474, NNF20OC0060610), ‘Fonden til lægevidenskabens fremme’, the Familien Hede Nielsen foundation and the Independent Research Fund Denmark (0134-00406A). E.B.J. was supported by Aarhus University and E.B.J. and C.H.G by the Independent Research Fund Denmark (2096-00165A). J.M.K. was supported by Lundbeckfonden (R307-2018-3667), Carlsberg Fonden (CF19-0687), Novo Nordisk Fonden (0073440) and Steno Diabetes Center Aarhus (SDCA). The authors declare no conflicts of interest.

**TRIAL REGISTRATION NUMBER:**

N/A.

## Introduction

Hypergonadotropic hypogonadism, small testes, and non-obstructive azoospermia (NOA), with the resultant endpoint being infertility, are some of the hallmarks of Klinefelter syndrome (47,XXY; KS). The testicular phenotype in males with KS is well described, including degeneration of germ cells, Leydig cell (LC) hyperplasia, hyalinization of the seminiferous tubules, thickening of the tubule walls, fibrosis, and in some cases spermatogenic foci (Sciurano *et al.*, 2009; Aksglaede and Juul, 2013; Gravholt *et al.*, 2018). However, the exact cause of testicular degeneration and hypogonadism remains poorly understood.

Infertility affects ∼2.5–12% of the male population (Agarwal *et al.*, 2015), with a heterogeneous phenotypic presentation ranging from decreased sperm quality as observed in cryptozoospermia (CO) (Abhyankar *et al.*, 2016) to complete absence of spermatozoa in the ejaculate, termed azoospermia. Azoospermia, found in 1% of the non-vasectomized male population, is usually divided into obstructive azoospermia (OA, 25–40%) and NOA (60%-75%) (Jarow *et al.*, 1989; Fedder *et al.*, 2004). While NOA largely comprises idiopathic cases (iNOA), a genetic etiology is found in approximately 15% of infertile men and in 30% of males with azoospermia (Dohle *et al.*, 2002; Fedder *et al.*, 2021). Major genetic causes of NOA include chromosomal anomalies, with the most frequent being KS and microdeletions in the azoospermia factor (AZF) region of the Y chromosome. While some of the abovementioned features of KS are syndrome specific, others are shared with other subgroups of NOA (e.g., Sertoli cell (SC)-only tubules). The molecular background for the testicular phenotypes in subgroups of NOA, such as KS, has not been elucidated.

With the rapid advancement of high-resolution genomic techniques, such as single-cell RNA sequencing (scRNAseq), the understanding of the molecular mechanisms associated with the testicular phenotype of NOA has improved (Wang *et al.*, 2018; Laurentino *et al.*, 2019; Zhao *et al.*, 2020; Alfano *et al.*, 2021; Mahyari *et al.*, 2021; Di Persio *et al.*, 2021; Chen *et al.*, 2022). Collectively, they demonstrate an altered cellular composition in the testes, with immature LCs and SCs and enrichment of pro-inflammatory macrophages in both males with iNOA and KS (Di Persio and Neuhaus, 2023). In addition, these studies reveal that SCs in males with KS display a distinct profile with higher expression of immune response-related and X-linked genes, pointing towards a significant role of the immune system in the testicular pathogenesis of NOA, with a unique profile in KS.

Recent reports have indicated the presence of microvascular dysfunction in the testes of males with KS (Van Saen *et al.*, 2018). Intriguingly, one study found increased levels of intratesticular testosterone in males with KS, suggesting a deficient release into the systemic circulation which could be responsible for the hypogonadism seen in these patients (Tuttelmann *et al.*, 2014). In addition, the same group showed that the testicular microvasculature was dysfunctional in a KS mouse model, where adult male 41,XX^y^ mice had an increased density of blood vessels (blood vessel area/testis volume) compared to 40,XY mice (Wistuba *et al.*, 2020). Similar changes were seen in testes from humans with increased vessel density in pre-pubertal boys with KS, driven by an increase in the number of smaller vessels (0–100 µm^2^) (Willems *et al.*, 2020). Although the differences in vascular density diminished over time, a tendency towards higher density of smaller vessels was still observed in adult males with KS. Furthermore, slower testicular blood perfusion kinetics and slower blood flow were associated with lower testosterone blood levels (Carlomagno *et al.*, 2022). These differences may be the result of a unique molecular profile of the microvasculature in males with KS, which could contribute to the hypogonadism and infertility observed in males with KS.

The testicular microvasculature, composed of endothelial cells (ECs), pericytes, and smooth muscle cells (SMCs), plays a crucial role in supporting both spermatogenesis and testosterone production. It supplies oxygen and nutrients to the seminiferous tubules and LCs, removes waste products, and exports testosterone into the circulation (Makela *et al.*, 2019). Given the increasing evidence suggesting that microvascular dysfunction may contribute to the testicular dysfunction in males with KS, this study aimed to explore the genomic landscape of the testicular microvasculature. Our goal was to demonstrate that by analyzing scRNAseq data from testicular cells of males with KS, we could identify microvascular cell types and investigate changes within these cell types. To overcome some of the inherent limitations associated with small sample sizes in individual scRNAseq studies, we collected publicly available scRNAseq datasets of human testicular samples, creating a larger cohort with a greater number of cells compared to previous individual studies. We conducted a comprehensive analysis of the gene expression of the various cell types within the testicular vasculature to gain deeper insights into the potential molecular mechanisms underlying testicular vasculature development and maintenance in males with KS and other subgroups of male infertility. Our findings aimed to elucidate the gene expression profile of the microvasculature in KS, shedding light on a possible link between altered gene expression in capillary ECs and a dysfunctional microvasculature.

## Materials and methods

### Sample inclusion

Samples were included from six publicly available scRNAseq studies of testicular tissue from adult males (Guo *et al.*, 2018; Laurentino *et al.*, 2019; Zhao *et al.*, 2020; Mahyari *et al.*, 2021; Di Persio *et al.*, 2021; Nie *et al.*, 2022). Combined, these comprised data from 29 males, including six with KS, four with iNOA, one patient with an AZF microdeletion (AZFdel), one patient with OA, 14 controls (CTRL), and three with CO. Males with AZFdel and iNOA were combined in a NOA (non-KS) group. Since we would expect a normal testicular phenotype in the male with OA, this sample was added to the control group.

### Sample processing

Three control samples (CTRL2–4) and three iNOA samples (iNOA2–4) were generated from the BD Rhapsody platform, referred to as ‘BD samples’, while the remaining samples were generated from the 10x Genomics platform, referred to as ‘10× samples’. For the BD samples, gene expression count files were downloaded. For 10x samples, fastq-files were downloaded and demultiplexed via CellRanger (10X Genomics Cell Ranger 6.1.1, CA, USA). Seurat objects were created using the Seurat package in R (Seurat 4.1.1) (Hao *et al.*, 2021). Prior to normalization, scaling, and clustering, objects were filtered to <10% mitochondrial genes, expression of 200–10,000 genes and a UMI-count (the absolute number of unique RNA molecules) below 150,000. The initial cell clustering appeared diffuse with some overlapping clusters (Supplementary Fig. S1A). Further analysis revealed substantial contamination in all clusters by the spermatozoa-specific genes *PRM1* and *PRM2* (Supplementary Fig. S1C), which was particularly evident in the control samples, while absent in the NOA and KS samples (Supplementary Fig. S1E). To address this issue, we implemented the SoupX algorithm (SoupX 1.6.0) (Young and Behjati, 2020), to remove cell-free RNA contamination. The soupX algorithm was applied using an automatic contamination fraction estimate to adjust the counting profile. This step reduced most of the contamination and resulted in a better separation between the individual clusters (Supplementary Fig. S1B, D, and F). We observed a well-integrated dataset when scrutinizing the individual samples, groups, and studies (Supplementary Fig. S2A, C, and D), and a clear separation between spermatogenic and somatic cells (Supplementary Fig. S2B). DoubletFinder was applied to each sample separately to remove duplicated cells (DoubletFinder 2.0.3) (McGinnis *et al.*, 2019), starting with optimal pK value selection and homotypic doublet proportion estimation, adding a singlet/doublet call to each cell of the Seurat object. Six samples (KS5–6, iNOA1, CTRL5–7) had been run in duplicate (.1 and .2 suffix) and were merged prior to data integration, performed with the IntegrateData function of the Seurat package. Briefly, this function detects matching cell pairs across different datasets. These pairs help in addressing technical variations between datasets and enable comparative analysis across experimental conditions.

### Cell type annotation

Testicular cell types were determined based on marker genes obtained from the literature. From here, EC and SMC clusters were isolated. Cells expressing the spermatogenic markers *PRM1*, *PRM2*, and *TNP1* > 2 were removed to avoid spermatozoa contamination in the subsequent analyses. Vascular EC types were subcategorized as arterial, venous, capillary, or lymphatic based on a murine EC single-cell atlas by Kalucka *et al.* (2020), from which testicular markers were used.

### Differential expression and Gene Ontology

Differential expression analysis was carried out for each vascular cell type via the FindMarkers function of the Seurat package, for KS versus controls, NOA versus controls, and KS versus NOA. Significance was established as an adjusted *P*-value of 0.05 (*P*_adj_ < 0.05) and a log2 fold change of 0.5, unless stated otherwise. To avoid residual spermatozoa contamination and dead cells affecting the analyses, mitochondrial genes/*PRM1*/*PRM2*/*TNP1* were excluded from the differential expression output. To examine gene expression in specific Gene Ontology (GO) categories, tables of genes within terms of interest (angiogenesis, GO:0001525; tight junctions, GO:0070160) were obtained from QuickGO (ebi.ac.uk/QuickGO), selecting taxon ‘Homo sapiens’ (9606). The FindMarkers function was applied and restricted to the capillary EC cells and the genes within each selected GO term. Adjusted *P*-values and log2 fold changes were extracted to generate plots displaying the expression differences between KS, NOA, and CTRL using ggplot2 (Ginestet, 2011). Only genes that were differentially expressed in the KS versus CTRL comparison were included in the plot.

### Cellular communication analysis

Cellular interaction and communication patterns were analyzed via CellChat (CellChat 1.6.1) (Jin *et al.*, 2021), using a human database of cell–cell communication. This was performed for all testicular cell types and for subgroups of vascular cell types. The three sample groups, KS, NOA, and CTRL, were compared pairwise, identifying overexpressed genes, communication probabilities, signaling pathways, and cellular communication networks. Dominant senders and receivers were analyzed in the 2D space. For lymphocytes and capillary ECs, incoming and outgoing signaling networks were ranked based on information flow. In addition, significant interactions between lymphocytes and ECs, that were increased in KS, were analyzed.

### Statistical analyses

Differences in cell type frequency between groups were analyzed using a Kruskal–Wallis test. Pairwise comparisons were then performed using a Wilcoxon rank sum test. Results were considered statistically significant when *P* < 0.05 or *P*_adj_ < 0.05 when multiple hypothesis correction was applied. All statistics were calculated using the R software (R 4.2.1, Vienna, Austria).

## Results

### Sample inclusion and analysis workflow

**Figure 1.**
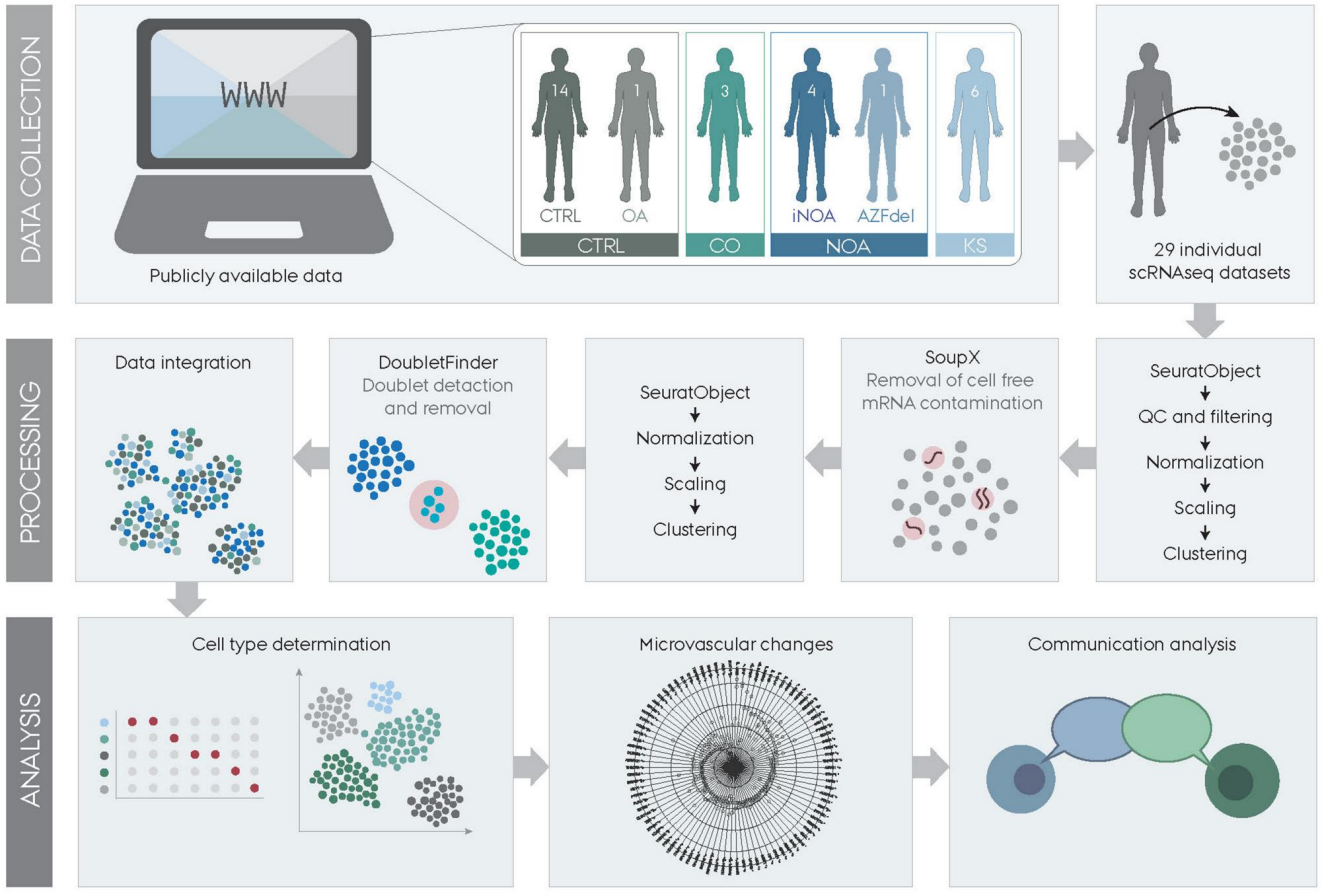
**A schematic illustration of the study workflow for the analysis of the testicular microvasculature in males with Klinefelter syndrome.** A total of 29 publicly available single-cell RNA sequencing datasets were downloaded, consisting of 15 control males (CTRL), 3 males with cryptozoospermia (CO), 5 males with non-obstructive azoospermia (NOA), and 6 males with Klinefelter Syndrome (KS). Quality control, filtering, normalization, and scaling were performed on each sample before applying SoupX to eliminate cell-free mRNA contamination and DoubletFinder to detect and remove doublets. The individual datasets were then integrated, and the testis cell types were annotated based on expression of cell-type-specific markers obtained from the literature. We used the integrated dataset to investigate the testicular microvascular changes and cell–cell communication.

To investigate the testicular microvasculature of males with KS at the cellular level, we included samples from six publicly available scRNAseq studies of testicular tissue from adult males (Guo *et al.*, 2018; Laurentino *et al.*, 2019; Zhao *et al.*, 2020; Mahyari *et al.*, 2021; Di Persio *et al.*, 2021; Nie *et al.*, 2022). We divided the samples into four groups based on the reproductive conditions of the individuals: males with KS, males with NOA (iNOA, AZFdel), controls (CTRL, OA), and males with CO (Fig. 1, upper panel). The general workflow for the analysis of these groups was performed as outlined (Fig. 1, middle and lower panel). In brief, we processed each sample individually, removed contaminating cell-free RNA and doublets, and then integrated all samples. Through clustering analysis of the integrated dataset, we identified the various cell types within the testis and sub-classified the ECs to identify microvascular ECs. Finally, we analyzed the changes in the testicular microvasculature of males with KS.

### Clustering and cell type annotation

**Figure 2.**
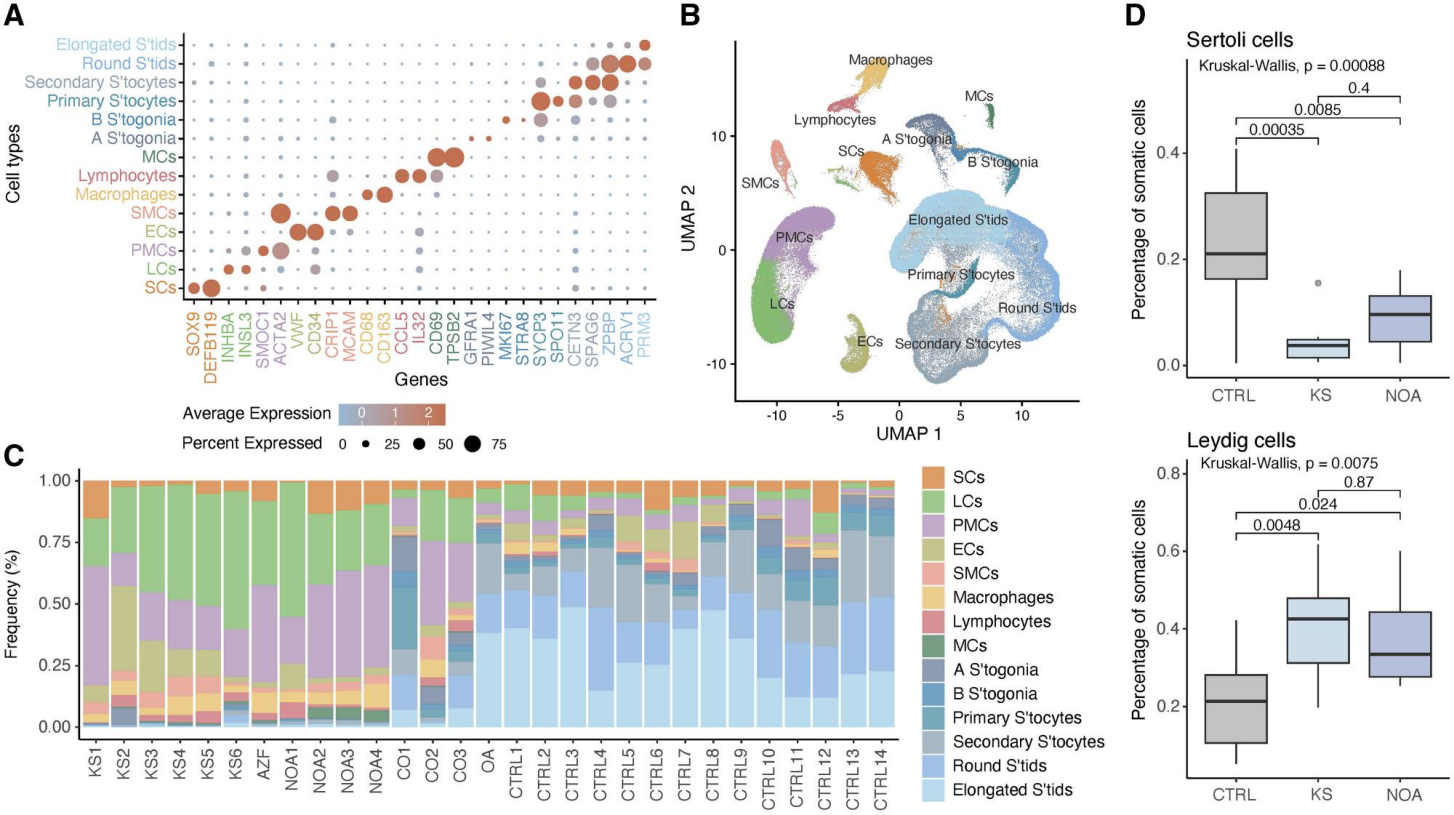
**Annotation of the different testicular cell types.** (**A**) Dotplot displaying the expression of cell-type-specific marker genes used to identify testicular cell populations. The color and size of each dot represent the average expression and the percentage of expressing cells, respectively, within the given cell type cluster. (**B**) UMAP of all testicular cell populations from the 29 males. Six spermatogenic clusters and eight somatic clusters were identified. (**C**) Frequency distribution of cell types within each individual. (**D**) Relative somatic counts of Sertoli (top) and Leydig (bottom) cells. KS, Klinefelter syndrome; AZF, azoospermia factor; NOA, non-obstructive azoospermia; OA, obstructive azoospermia; CTRL, control; SCs, Sertoli cells; LCs, Leydig cells; PMCs, peritubular myoid cells; ECs, endothelial cells; SMCs, smooth muscle cells; MCs, mast cells; S’togonia, spermatogonia; S’tocytes, spermatocytes; S’tids, spermatids.

In the integrated dataset, we identified and annotated 14 distinct cell clusters based on the expression of known cell type-specific markers (Fig. 2A and B). Five clusters of spermatogenic cells (spermatogonia, primary and secondary spermatocytes, round and elongated spermatids) were distinguished, while eight clusters of somatic cells were identified (ECs, mast cells (MCs), macrophages, lymphocytes, SCs, SMCs, peritubular myoid cells and LCs). The top markers for each cell cluster were determined (Supplementary Fig. S3). Pronounced variations in the cellular quantities among individuals were observed. As expected, very few spermatogenic cells were observed in the males with KS and NOA (Fig. 2C). Excluding these spermatogenic cells, a higher relative somatic count of SCs was present in controls compared to males with NOA (*P* = 0.0085) and KS (*P* = 0.00035), which in turn had higher relative somatic counts of LCs compared to controls (*P* = 0.0048 KS; *P* = 0.024 NOA) (Fig. 2D).

### Identifying cell types of the testicular microvasculature

**Figure 3.**
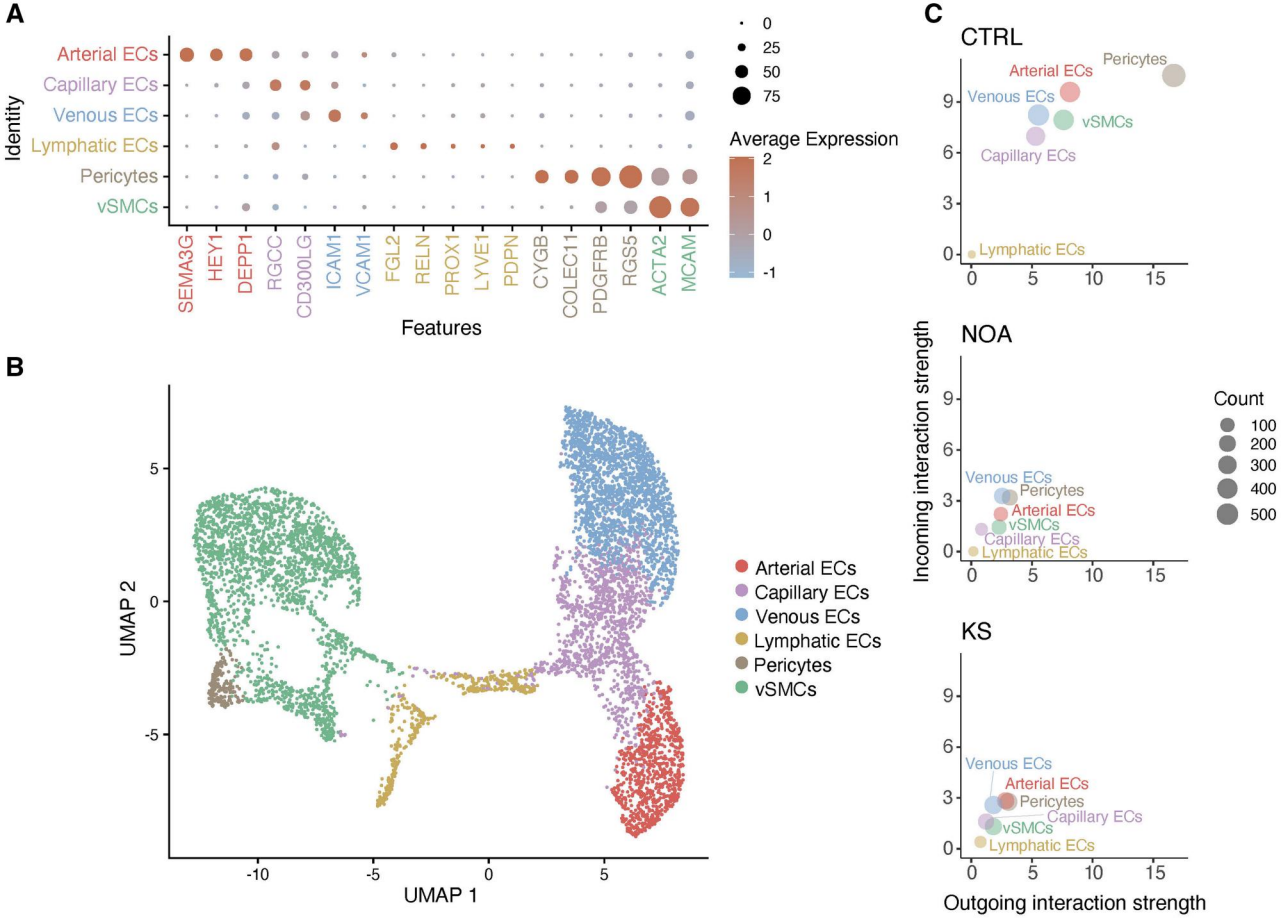
**Testicular microvasculature cell sub-populations.** (**A**) Dotplot displaying the expression of cell-type-specific marker genes used to identify testicular ECs and smooth muscle cell sub-populations. The color and size of each dot represent the average expression and the percentage of expressing cells, respectively, within the given cell type cluster. (**B**) A UMAP plot of the testicular EC sub-populations (arterial ECs, capillary ECs, venous ECs, and lymphatic ECs) and smooth muscle cell sub-populations (vSMCs and pericytes). (**C**) Cell signaling was assessed as incoming and outgoing interaction strength for each EC and smooth muscle cell sub-population within control (CTRL, top), non-obstructive azoospermia (NOA, middle), and males with Klinefelter syndrome (KS, bottom). The dot size indicates the interaction strength. Interaction strengths were based on the ligand–receptor pairs included in the CellChat algorithm (see Material and Methods section). ECs, endothelial cells; vSMC, vascular smooth muscle cells.

To identify the cell types of the testis mostly responsible for oxygen, nutrient, and hormone exchange, i.e. the cell types that constitute the microvasculature, we filtered the collected dataset to include only ECs and SMCs and performed re-clustering. Based on the testis EC markers described by Kalucka *et al.* (2020), we identified arterial, capillary, venous, and lymphatic ECs, vascular SMCs, and pericytes (PCs) (Fig. 3A and B). The top markers for each cell cluster were determined (Supplementary Fig. S4). Unfortunately, the males with CO had a small number of cells within each sub-population (Supplementary Fig. S5); therefore, we removed the males with CO from further analysis owing to the lack of statistical power within this group.

### Vascular cell-to-cell communication is impaired in males with KS and NOA

First, we conducted an overall analysis of genes associated with cell-to-cell communication among all the vasculature cell types. Notably, we found that expression of genes associated with cell-to-cell communication was stronger in controls, for all cell types, compared to males with KS and NOA, suggesting an overall impaired or dysfunctional vasculature in males with KS and NOA (Fig. 3C).

### Capillary ECs in males with KS show an altered gene expression profile

**Figure 4.**
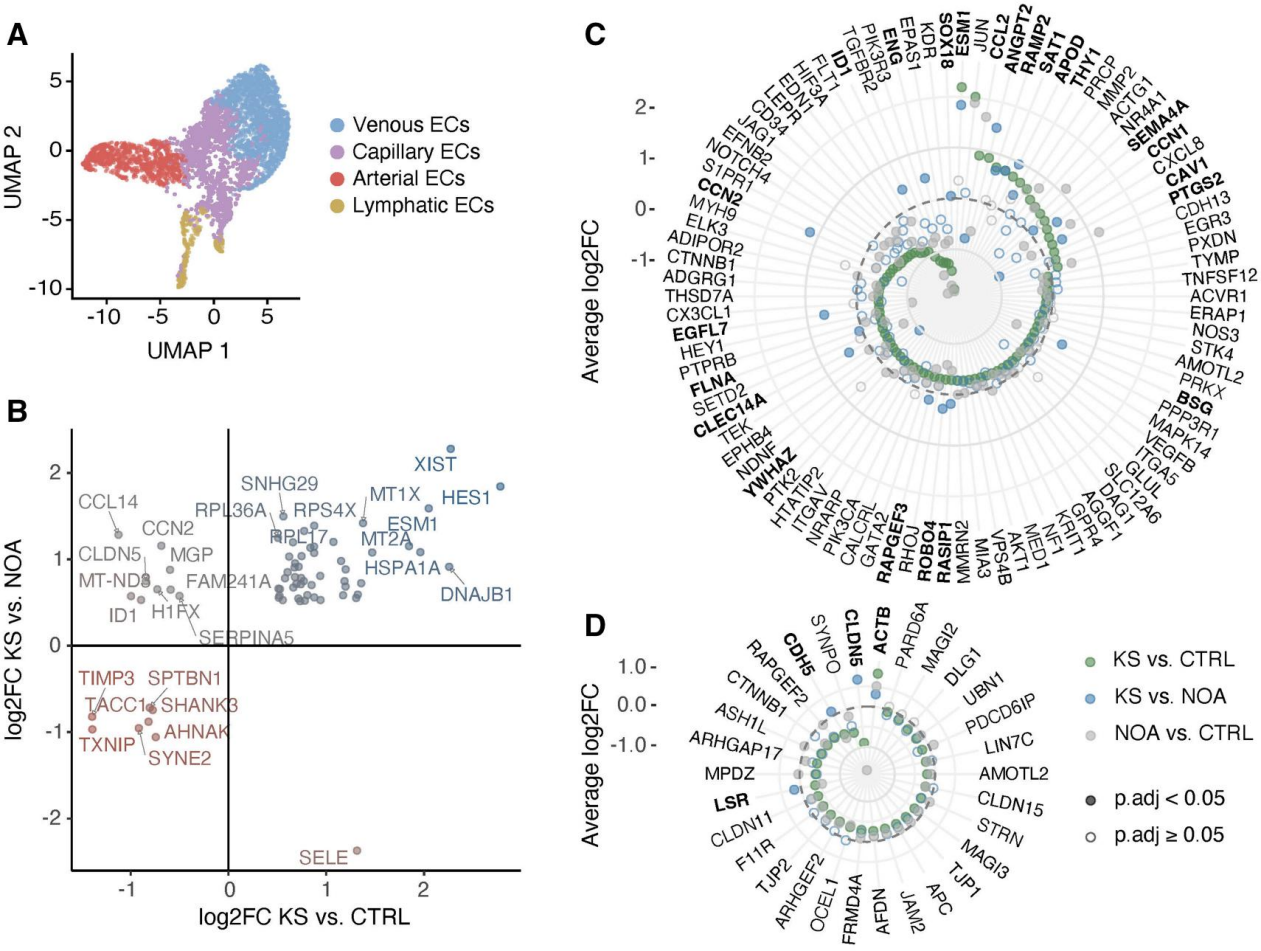
**Differential expression in capillary endothelial cells and changes related to angiogenesis and barrier function.** (**A**) UMAP plot of the endothelial cell (EC) sub-populations: arterial ECs, capillary ECs, venous ECs, and lymphatic ECs. (**B**) Differentially expressed genes in capillary ECs in Klinefelter syndrome (KS) versus controls (CTRL) (*x*-axis) and in KS versus non-obstructive azoospermia (NOA) (*y*-axis) (*P*_adj_ < 0.05). Average difference in expression in capillary ECs between KS and CTRL (green), KS and NOA (blue), and NOA and CTRL (grey) for genes within Gene Ontology terms for angiogenesis (**C**) and tight junctions (**D**). Differentially expressed genes are marked by filled circles. Gene names in bold indicate differential expression in both KS versus CTRL and KS versus NOA.

We then examined the microvascular cell types found in capillaries; capillary ECs and PCs. Only a few PCs were found in each group and, consequently, we excluded PCs from the downstream analysis and focused on capillary ECs to investigate possible gene expression changes in males with KS (Supplementary Fig. S5). By comparing males with KS to both males with NOA and CTRLs, we identified differentially expressed genes in KS capillary ECs (*P*_adj_ < 0.05) (Fig. 4B). As expected, *XIST* was upregulated in males with KS compared to both males with NOA and CTRLs. Additionally, we found a strong upregulation of *ESM1* and *HES1*, and a considerable downregulation of *TIMP3*, *SYNE2*, and *AHNAK* (Fig. 4B). Previous studies have demonstrated the involvement of these genes in vascular remodeling, such as EC activation and angiogenesis initiation (*ESM1*, *HES1*, *TIMP3*) and EC barrier tightness (*ESM1*, *TIMP3*, *AHNAK*) (Gentil *et al.*, 2005; Kitagawa *et al.*, 2013; Rocha *et al.*, 2014; Fan and Kassiri, 2020) (see Discussion).

### Microvascular changes in males with KS are associated with EC activation and barrier function

To gain further insight, we investigated changes in genes involved in angiogenesis (GO:0001525) (Fig. 4C). Only genes within this ontology that were differentially expressed in KS versus CTRLs were included. Among 19 upregulated genes, 11 (58%) were also upregulated in males with KS compared to males with NOA and hence uniquely upregulated in males with KS. These included *ANGPT2*, *APOD*, *CAV1*, *CCL2*, *CCN1*, *ESM1*, *PTGS2*, *RAMP2*, *SAT1*, *SEMA4A*, and *THY1*, pointing toward EC inflammation and activation. Conversely, 11 out of 69 (16%) downregulated genes compared to CTRLs were also downregulated compared to males with NOA, and hence uniquely downregulated in males with KS. These included *CCN2*, *CLEC14*, *EGFL7*, *ENG*, *FLNA*, *ID1*, *RAPGEF3*, *RASIP1*, *ROBO4*, *SOX18*, and *YWHAZ*, indicating reduced signaling in capillary ECs in both males with KS and NOA. This observation was supported by the amount of outgoing and incoming signaling in capillary ECs of males with KS (Supplementary Fig. S6).

We also studied genes associated with EC barrier function, specifically tight junctions (Fig. 4D) (GO:0070160). Overall, we observed a downregulation of most EC tight junction genes in both males with KS and NOA, including a strong downregulation of *CLDN5* and *CDH5 (VE-Cadherin)*, which play crucial roles in EC barrier tightness (Richards *et al.*, 2022). Only one gene, *ACTB*, was upregulated, a gene previously shown to be involved in EC remodeling (Dugina *et al.*, 2021).

### Increased lymphocyte activation is specific to males with KS

**Figure 5.**
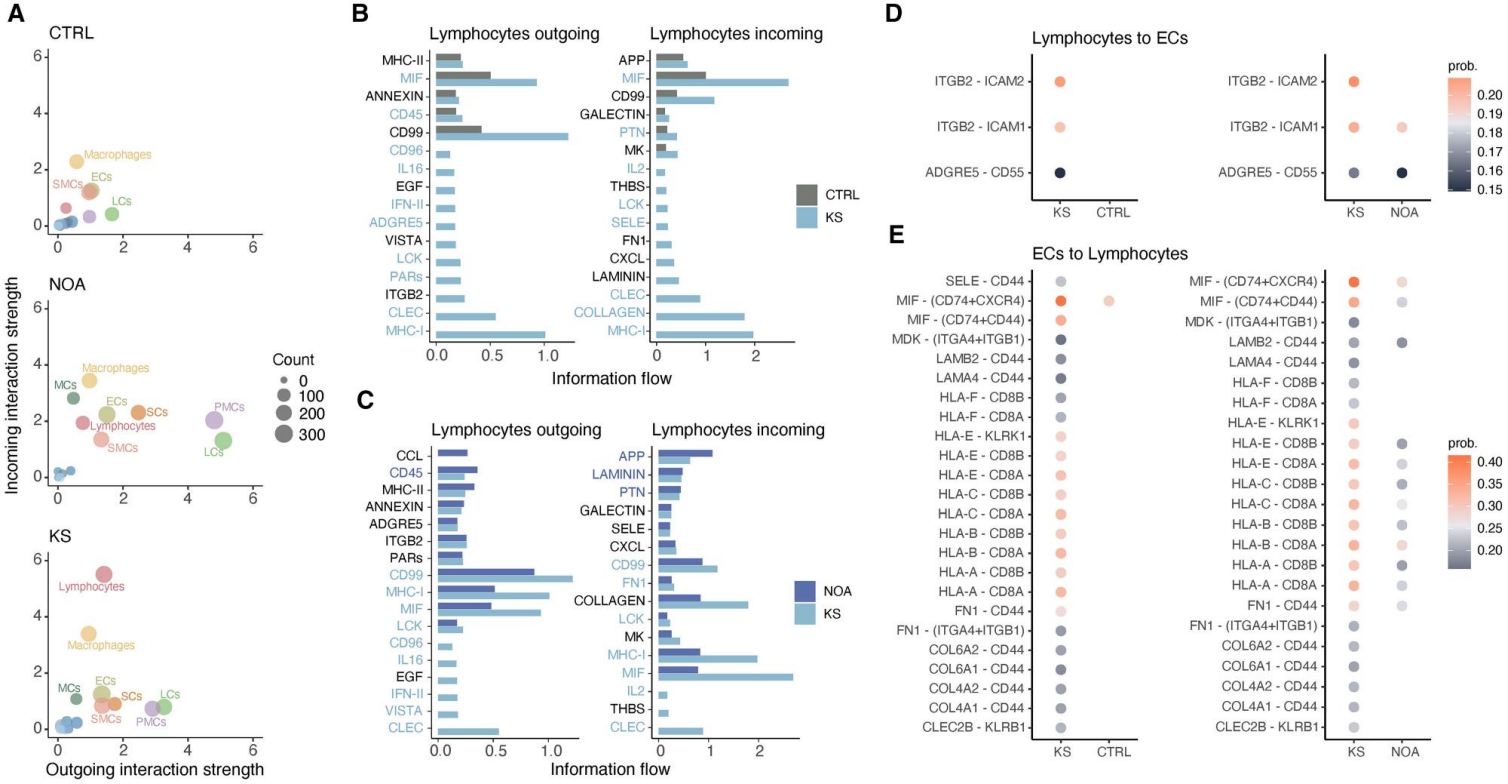
**Cell–cell communication of the testicular cell types.** (**A**) Incoming and outgoing interaction strength for each testicular cell type obtained from control males (CTRL, top), males with non-obstructive azoospermia (NOA, middle), and males with Klinefelter syndrome (KS, bottom). The dot size is proportional to the strength of the interaction, which was calculated using the CellChat algorithm. (**B**–**C**) The outgoing (left) and incoming (right) information flow for lymphocytes when comparing KS to CTRL (**B**) and KS to NOA (**C**). The light blue markings highlight enriched signaling pathways and the unique communication pattern in KS. (**D**–**E**) Significant interactions (*P* < 0.01) increased in males with KS compared to CTRLs (left) and males with NOA (right) from lymphocytes to ECs (**D**) and ECs to lymphocytes (**E**). The color indicates the communication probability calculated using the CellChat algorithm. SCs, Sertoli cells; LCs, Leydig cells; PMCs, peritubular myoid cells; ECs, endothelial cells; SMCs, smooth muscle cells; MCs, mast cells.

Expanding upon the findings above, we investigated the possible association between dysfunctional EC behavior and inflammation, by analyzing cell signaling in the different cell types in males with KS, males with NOA, and CTRLs. Based on gene expression, our results showed increased macrophage signaling in males with KS and NOA compared to CTRLs, in addition to a markedly increased lymphocyte signaling, especially incoming, in males with KS (Fig. 5A). Expression of genes associated with pro-inflammatory signaling pathways, such as MIF, MHC-I, CLEC and CD99, were upregulated in lymphocytes from males with KS compared to lymphocytes from both males with NOA and CTRLs (Fig. 5B and C). In addition, upregulation of genes associated with signaling between ECs and lymphocytes involving ligand-receptor interactions, such as ITGB2-ICAM, MIF-CD74, and MHC-I, was seen (Fig. 5D and E). Overall, the observed EC remodeling in males with KS may be driven by a pro-inflammatory environment in addition to inflammation in the ECs themselves.

**Figure 6.**
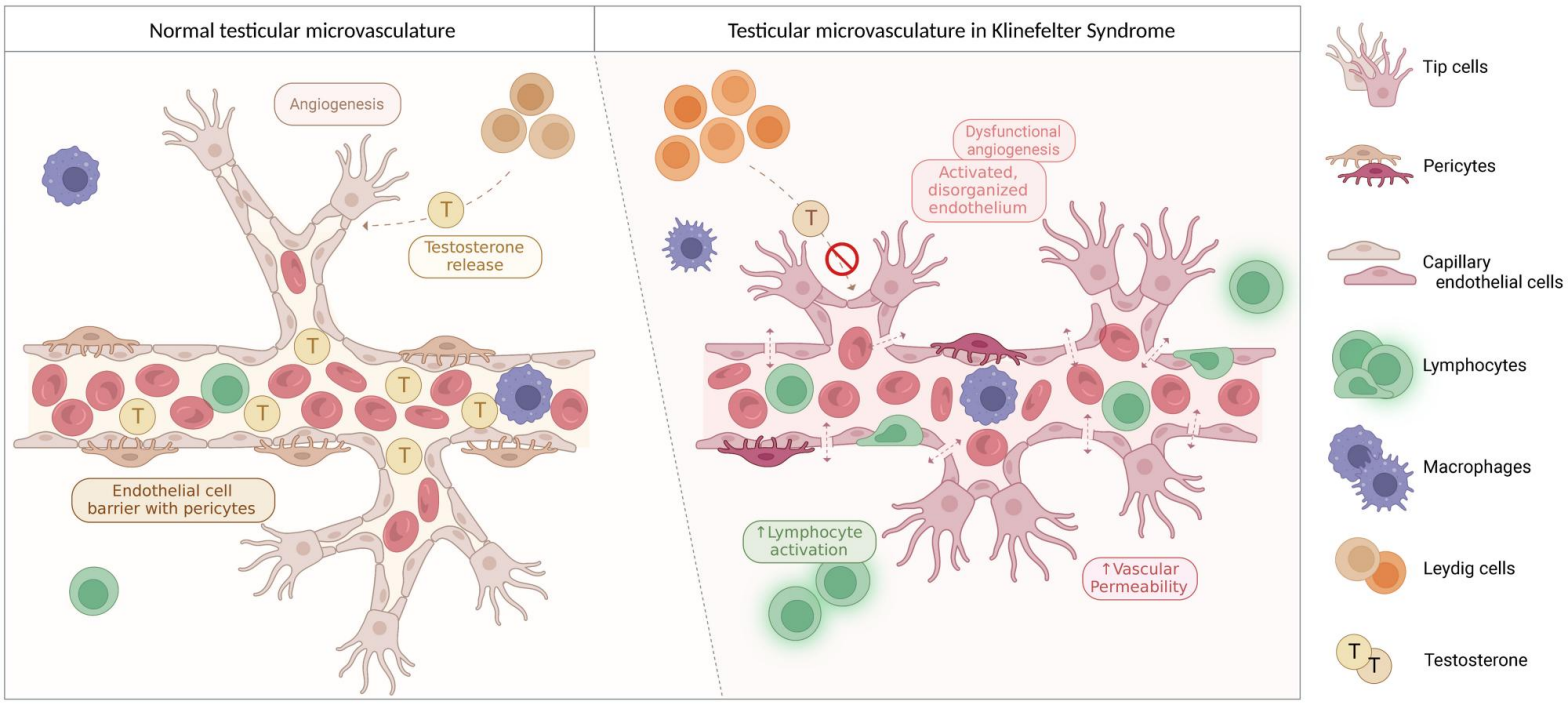
**Illustration of the proposed altered testicular microvasculature in Klinefelter syndrome.** In healthy male controls, the testicular microvasculature is maintained by angiogenesis leading to mature capillaries responsible for oxygen, nutrient, and hormone exchange. The microvasculature consists of a capillary network lined with ECs with pericytes embedded in the basement membrane. Lymphocytes can adhere and migrate across (left). We propose that the microvasculature in Klinefelter syndrome is characterized by excessive disorganized vessel formation leading to immature vessels with compromised vascular integrity, associated with increased lymphocyte recruitment and activation. This may result in a decreased ability to shuttle testosterone into the blood, leading to decreased circulating testosterone levels (right). ECs, endothelial cells.

Taken together, these findings suggest that the testicular vasculature in males with KS is characterized by EC activation and altered EC homeostasis, independent of the traditional angiogenic pathways, and possibly mediated by increased inflammation, leading to immature vessels with compromised integrity (Fig. 6).

## Discussion

Our understanding of KS and the associated hypogonadism and infertility has seen advances in recent years, partially owing to the employment of high-resolution genomic techniques such as scRNAseq. Moreover, observations of microvascular changes within the testes of males with KS have further contributed to this process. Our study builds on these insights by elucidating the specific microvascular alterations within the testes of males with KS. Notably, we report a unique and distinct gene expression profile of capillary ECs in males with KS, characterized by upregulation of genes associated with inflammation and EC activation, and downregulation of genes associated with vessel maturation, barrier tightness, and integrity. These findings suggest a possible role of microvascular dysfunction in the testis pathophysiology observed in males with KS (Fig. 6).

In the present study, we collected and re-analyzed publicly available datasets of single-cell populations from testicular tissue and showed that components involved in normal function of the vasculature are dysregulated in males with KS (Guo *et al.*, 2018; Laurentino *et al.*, 2019; Zhao *et al.*, 2020; Mahyari *et al.*, 2021; Di Persio *et al.*, 2021; Nie *et al.*, 2022) (Fig. 6). The present results seem to fit well with previous results in both humans and mice, showing microvascular dysfunction in both model systems reported as slower blood flow and an increased density of smaller blood vessels (Willems *et al.*, 2020; Wistuba *et al.*, 2020; Carlomagno *et al.*, 2022). Based on this, our research provides new insights into the possible interplay between a dysfunctional microvasculature and the testicular crisis leading to hypogonadism and infertility in males with KS.

The upregulated genes identified in males with KS support the idea of EC activation and vessel destabilization in the testicular microvasculature of males with KS. Three of these genes, *ESM1*, *APOD*, and *CCN1*, were associated with an enrichment in EC tip cells, which are the guiding and most immature cells during angiogenesis initiation (Rocha *et al.*, 2014; Park *et al.*, 2019; Zarkada *et al.*, 2021). Upregulation of *THY1*, *CCL2*, and *PTGS2* indicated an increased inflammation leading to increased EC activation (Lee *et al.*, 1998; Ridiandries *et al.*, 2016), whereas upregulation of *ESM1* and *ANGPT2* could lead to immature vessels with increased vascular permeability (Rocha *et al.*, 2014; Akwii *et al.*, 2019). On the other hand, we observed downregulation of hallmark genes normally associated with vessel maturation, including *KDR (VEGFR2)*, *ENG*, *NOTCH4*, *JAG1*, and *ID1*, in addition to the anti-angiogenic gene *FLT-1 (VEGFR1)* (Muhleder *et al.*, 2021). Thus, the increased EC activation seemed to be independent of the traditional pathways and, instead, mediated by increased inflammation (discussed below), leading to dysfunctional EC activation, and possibly angiogenesis initiation, with vessels that do not fully mature. Notably, *SOX18* was strongly downregulated in both males with KS and NOA. Previously, *SOX18* has been shown to be important for the formation of the EC barrier by increasing the levels of claudin 5, a junctional protein, encoded by *CLDN5* (Fontijn *et al.*, 2008). The connection between *SOX18* and *CLDN5* was also obvious in our data, as *CLDN5* was significantly downregulated in males with KS. Claudin 5 has been shown to play a crucial role in maintaining EC barrier integrity, with its loss leading to decreased vascular integrity and altered junction composition (Richards *et al.*, 2022). Furthermore, when looking at both incoming and outgoing communication in the capillary ECs, based on gene expression, it was evident that most pathways needed for normal angiogenesis and vessel maturation were downregulated in males with KS compared to CTRLs (Supplementary Fig. S6).

The observed characteristics of activated and inflamed ECs leading to a compromised microvasculature in males with KS raise several questions, particularly regarding the implications for testosterone production and the mechanisms underlying testicular atrophy in KS. It may be that a disorganized microvasculature with decreased integrity and increased inflammation hampers testosterone release. Considering that testosterone relies on a well-functioning vascular network for efficient synthesis and subsequent release into the blood stream, the dysfunctional microvasculature could provide a new perspective on testosterone deficiency in KS. This is supported by previous studies demonstrating a correlation between EC dysfunction, microvascular flow, and reduced systemic testosterone levels (Corrigan *et al.*, 2015; Carlomagno *et al.*, 2022). Combining this with the indications of increased intratesticular testosterone levels only further underlines that a compromised testicular vasculature could be the cause of low systemic testosterone levels (Tuttelmann *et al.*, 2014; Wistuba *et al.*, 2020).

In addition to changes in the vasculature and the blood–testis barrier (BTB), previous studies of KS testes have identified an increased number of immune cells and increased inflammation as the possible foundation for the observed testicular fibrosis (D'Aurora *et al.*, 2015; Willems *et al.*, 2020; Bradshaw *et al.*, 2023). Cell-specific changes in gene expression pointed towards an increased signaling interaction strength of both macrophages and MCs in KS and NOA compared to CTRLs, and a marked increase in the incoming lymphocyte interaction strength in KS. Increased gene expression association with cellular signaling pathways in the lymphocytes of KS testes included activators of immune cells, such as MHC-I, CD99, and CLEC, as well as the pro-inflammatory cytokine MIF. We also found gene expression associated with an increased signaling through collagens, which have previously been linked to the fibrosis, hyalinization, and destabilization of the BTB in KS testes (D'Aurora *et al.*, 2015). The suggested interactions between lymphocytes and ECs were also investigated. Here, we identified several interactions of increased strength in males with KS. In signaling from lymphocytes to ECs, the interaction strengths between the integrin *ITGB2 (LFA-1)* and *ICAM1* and -*2* were increased and are known to be associated with innate immune responses and leukocyte adhesion to the microvasculature (Dustin and Springer, 1991). The interaction *ITGB2–ICAM2* was of particular interest, as the adhesion molecule ICAM-2 is expressed at EC junctions and is associated with vessel remodeling (Huang *et al.*, 2005). Signaling from ECs to lymphocytes had increased strength through several interactions, including components of MHC-I to CD8 (peptide fragment display to CD8+ cytotoxic T cells), extracellular matrix components laminin, fibronectin and collagen to CD44 (a marker of memory T cells), MIF to CD74 (activation of CD4+ T cells) and MHC-I to KLRK1 and CLEC2B to KLRB1 (natural killer cell activation). Taken together, these suggested interactions indicate increased inflammatory cross-talk and increased EC activation, with increased adhesion of lymphocytes to the ECs and activation of T-cells.

Based on our results, it is pivotal to address the sequence of events. Does the dysfunctional microvasculature lead to oxidative stress, hypoxia, inflammation, and impaired testosterone release, or is the dysfunctional microvasculature a consequence of inflammation in the testes, already being in a pathophysiological state? Regardless of the sequence of events, these processes seem to be intricately linked. Chronic microvascular dysfunction might potentiate inflammation and, conversely, chronic inflammation may disrupt the normal progression of angiogenesis and vessel maturation. As such, the cross-talk between the endothelium and the surrounding tissues plays a crucial role in the regulation and co-ordination of the inflammatory response and, importantly, both these scenarios could negatively interfere with hormonal control in the testes.

The general picture emerging concerning the genomics of KS is that across tissues, multiple changes in the DNA methylome, transcriptome, and metabolome are present (Sharma *et al.*, 2015; Belling *et al.*, 2017; Skakkebæk *et al.*, 2018; Winge *et al.*, 2020; Johannsen *et al.*, 2022; Viuff *et al.*, 2023; Davis *et al.*, 2023) and we and others have hypothesized that organ-specific genomic changes underlie the specific pathology related to that organ. Thus, the changes we describe in the testes seem to nicely recapitulate the pathology of the testes in adult males with KS. However, what this study does not explain is how this end result is reached. Is the deficient vasculature the sole culprit, together with signaling from the immune system, which also seems to be implicated, or are other molecular cues involved? Only a temporal study of the testis from embryological life until adulthood is likely to resolve which molecular processes are responsible for initiating and propelling the observed devastating changes of testicular catastrophe.

While the utilization of scRNAseq from publicly available databases enables a larger cohort size leading to a more precise examination of gene expression profiles in specific testicular cell types, we acknowledge that the scope of our study design warrants caution in drawing definitive conclusions. While our findings indeed point towards a complex interplay encompassing altered cellular communication, vascular remodeling, and pro-inflammatory responses in KS testes (Fig. 6), it is important to underscore that this study may not definitively identify the mechanisms responsible for microvascular dysfunction. This is a recognized challenge often associated with scRNAseq studies, warranting cautious interpretation and recognition of the potential for refined insights through future research.

Taken together, our results indicate a complex interplay of altered cellular communication, vascular remodeling, and pro-inflammatory responses within the testes of males with KS (Fig. 6). Specifically, the unique gene expression alterations observed in vascular cells in males with KS suggest excessive disorganized vessel formation resulting in immature vessels with compromised integrity. Thus, this offers novel insights into the testicular pathophysiology in KS and underscores the potential contribution of these processes to the hypogonadism and infertility seen in these males. While this study aims to better understand the microvascular dysfunction in adult KS, the precise connections to testosterone deficiency and testicular atrophy remain to be fully elucidated.

## Supplementary Material

dead224_Supplementary_Figure_S1

dead224_Supplementary_Figure_S2

dead224_Supplementary_Figure_S3

dead224_Supplementary_Figure_S4

dead224_Supplementary_Figure_S5

dead224_Supplementary_Figure_S6

## Data Availability

Single-cell sequencing data can be found in NCBI GEO under accession numbers GSE149512 (Zhao *et al.*, 2020), GSE120508 (Guo *et al.*, 2018), GSE130151 (Laurentino *et al.*, 2019), GSE169062 (Mahyari *et al.*, 2021), GSE153947 (Di Persio *et al.*, 2021), and GSE182786 (Nie *et al.*, 2022).
