# Supplementary material for: The testicular microvasculature in Klinefelter syndrome is immature with compromised integrity and characterized by excessive inflammatory cross-talk
Source: Hum Reprod. 2023 Oct 31;38(12):2339–49. doi: 10.1093/humrep/dead224 (PMC10694403; doi:10.1093/humrep/dead224)
Supplement: dead224_Supplementary_Figure_S1 [file dead224_supplementary_figure_s1.pdf]

Human Reproduction, 2023, 1–1
https://doi.org/10.1093/humrep/dead224
Supplementary Data

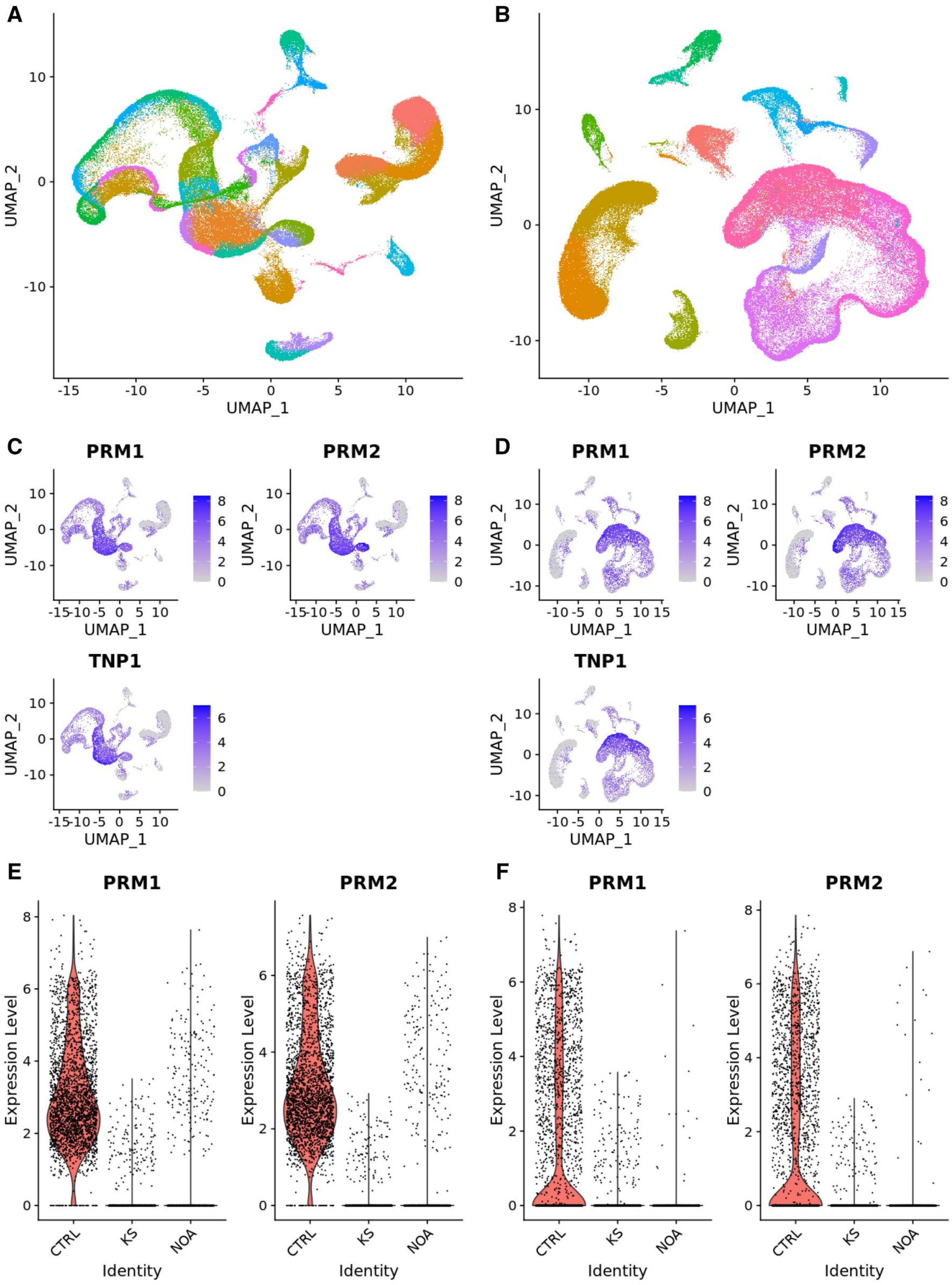

Supplementary Figure S1. Cell clustering and expression of spermatogenic and somatic markers. Initial clustering of all cells from the 29 included individuals, without (**A**) and with SoupX (**B**). Expression of spermatogenic markers *PRM1, PRM2* and *TNP1* in CTRL, NOA, and KS samples, without (**C**) and with SoupX (**D**). Expression of spermatogenic markers *PRM1* and *PRM2* in endothelial cells of CTRL, NOA, and KS samples, without (**E**) and with SoupX (**F**).
