# Supplementary figures and images for: The testicular microvasculature in Klinefelter syndrome is immature with compromised integrity and characterized by excessive inflammatory cross-talk
Source: Hum Reprod. 2023 Oct 31;38(12):2339–49. doi: 10.1093/humrep/dead224 (PMC10694403; doi:10.1093/humrep/dead224)

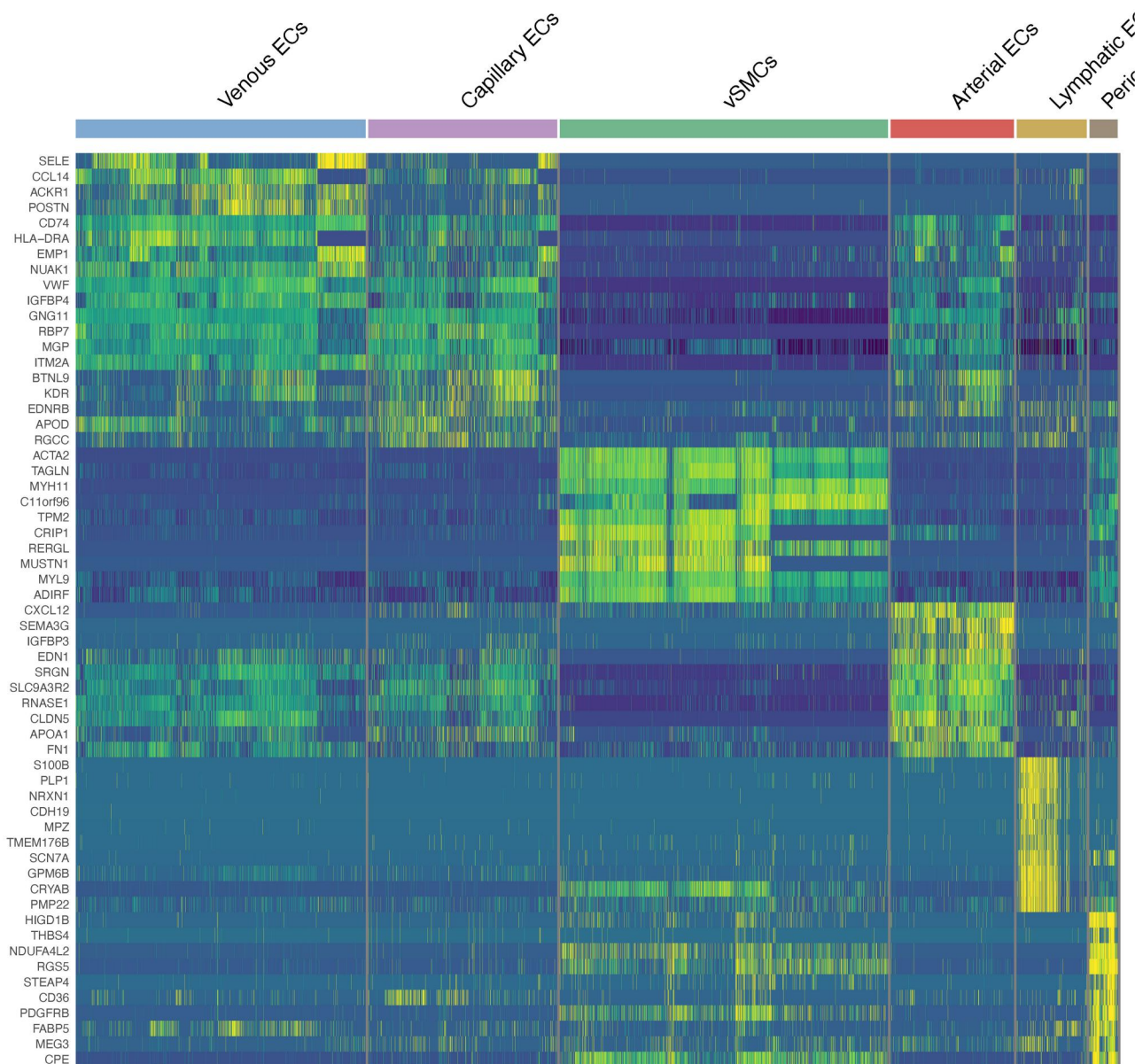

Supplementary Figure S4. Top 10 markers for each of the endothelial and muscle cell clusters.

Supplement: dead224_Supplementary_Figure_S4 [file dead224_supplementary_figure_s4.pdf]
