# Supplementary material for: The testicular microvasculature in Klinefelter syndrome is immature with compromised integrity and characterized by excessive inflammatory cross-talk
Source: Hum Reprod. 2023 Oct 31;38(12):2339–49. doi: 10.1093/humrep/dead224 (PMC10694403; doi:10.1093/humrep/dead224)
Supplement: dead224_Supplementary_Figure_S5 [file dead224_supplementary_figure_s5.pdf]

Human Reproduction, 2023, 1–1
https://doi.org/10.1093/humrep/dead224
Supplementary Data

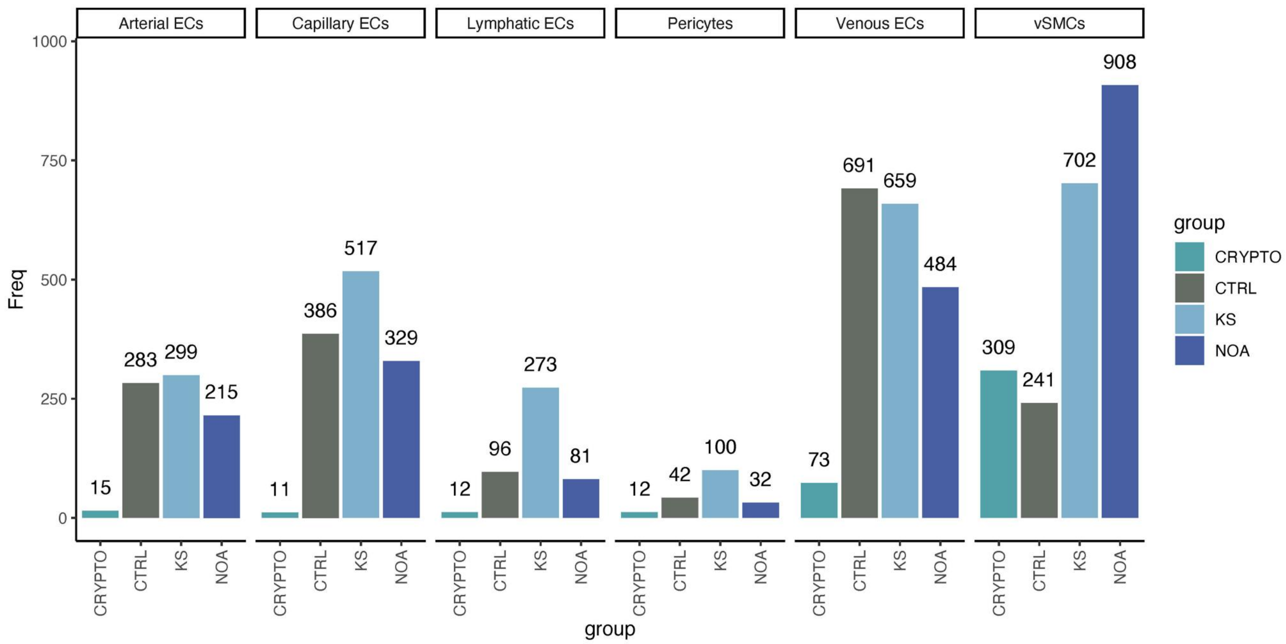

Supplementary Figure S5. Endothelial and smooth muscle cell subtype distributions across sample groups.
